# Supplementary material for: Strong charge carrier scattering at grain boundaries of PbTe caused by the collapse of metavalent bonding
Source: Nat Commun. 2023 Feb 9;14:719. doi: 10.1038/s41467-023-36415-1 (PMC9911745; doi:10.1038/s41467-023-36415-1)
Supplement: Supplementary file 1 — Supplementary Information [file 41467_2023_36415_MOESM1_ESM.pdf]

Supplementary Information

Strong Charge Carrier Scattering at Grain Boundaries of PbTe caused by the Collapse of Metavalent Bonding

Riga Wu,[1,#] Yuan Yu,[1,#,*] Shuo Jia,[1] Chongjian Zhou,[2] Oana Cojocaru-Mirédin,[1,*] Matthias Wuttig[1,3,*]

[1] Institute of Physics (IA), RWTH Aachen University, Sommerfeldstraße 14, 52074 Aachen, Germany

[2] State Key Laboratory of Solidification Processing, and Key Laboratory of Radiation Detection Materials and Devices, Ministry of Industry and Information Technology, Northwestern Polytechnical University, Xi'an 710072, China

[3] Peter Grünberg Institute (PGI 10), Forschungszentrum Jülich, 52428 Jülich, Germany

[#] equal contribution

[*] corresponding author

yu@physik.rwth-aachen.de (Y. Y.); cojocaru-miredin@physik.rwth-aachen.de (O. C.-M.); wuttig@physik.rwth-aachen.de (M. W.)

Supplementary Figures:

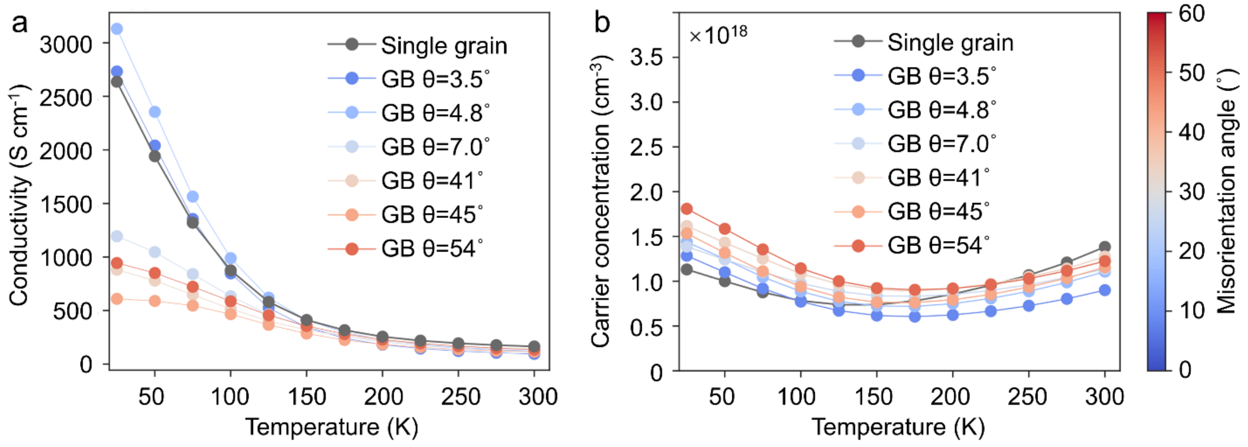

Fig. S1. Temperature-dependent (a) electrical conductivity and (b) Hall carrier concentration for samples with different GB misorientation angles. A single-grain sample is also measured for reference.

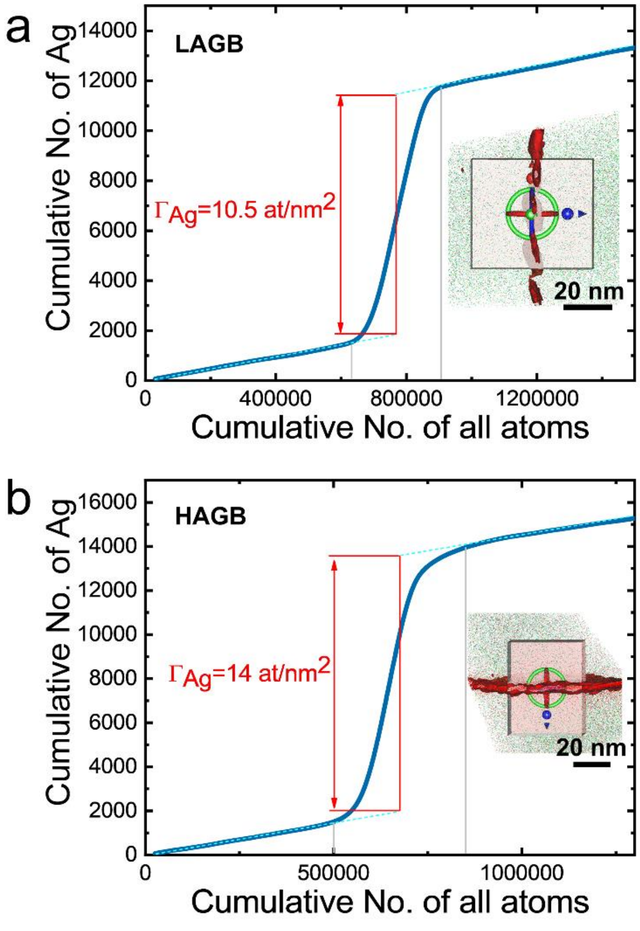

Fig. S2. Ladder diagram determined from the cuboid region of interest across the GBs. The Gibbsian interfacial excess of Ag at the GB can be calculated according to $\Gamma_{Ag} = N_{excess}/(\eta A)$, where

N_{excess} can be obtained from the ladder diagram indicated by the red arrow, A is the interfacial area of the selected region, and η is the APT detection efficiency (50% in case of LEAP 4000X Si). (a) LAGB; A=40 nm×45 nm. (b) HAGB; A=40 nm × 40 nm.

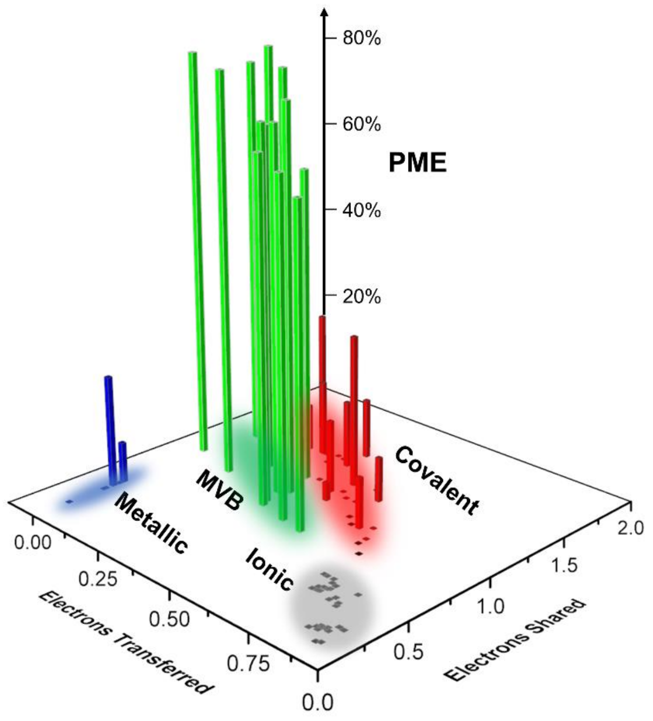

Fig. S3. PME value plotted on the basal plane of electrons transferred (ET) and electrons shared (ES). ET and ES are two natural variables describing the chemical bonding calculated by quantum chemical tools. More details about the ET-ES map can be found in Refs.[1, 2, 3]

In **Fig. S3**, a map is shown, where the PME value is shown for different compounds depicted in a map that has been developed recently. This map separates different bonding mechanisms based on material properties including the electrical conductivity σ, the Born effective charge Z* as a measure of the chemical bond polarizability, the effective coordination number, the optical dielectric constant ε_∞ as a measure of the electronic polarizability as well as the Grüneisen parameter as a measure of the bond anharmonicity. Different material classes have been classified based on unique sets of properties.[4] These materials can also be characterized by quantum-chemical bonding descriptors. As shown in Fig. S3, all metavalent compounds possess high PME values. Hence, there is clearly a correlation between metavalent bonding and bond breaking in the atom probe. We note in passing an interesting detail that is relevant in this context. Amorphous chalcogenides such as amorphous GeTe do not show large PME values and have the characteristic properties of covalently bonded solids.[5] This supports the idea that the bond rupture and the bonding mechanism are closely interwoven. Yet, the detailed mechanisms underpinning the high PME are still elusive due to the very complex evaporation process involving high electrical fields, surface states, band bending, and the interaction between specimen and laser, etc. We have found some possible correlations between the high PME and the large penetration depth of the electric field, which depends on the dielectric constant and effective masses of materials according to the Debye screening length for semiconductors. Interestingly, MVB compounds are characterized by large dielectric constants and small effective masses, leading to a large penetration depth of the

electric field. In addition, metavalent bonding is a kind of soft bond, which could be more unstable under high fields and laser excitations and, thus, several bonds break under one laser pulse generating multiple events.

References:

1. Raty JY, Schumacher M, Golub P, Deringer VL, Gatti C, Wuttig M. A Quantum-Mechanical Map for Bonding and Properties in Solids. *Adv Mater* **31**, 1806280 (2019).

2. Cheng Y, *et al.* Understanding the Structure and Properties of Sesqui-Chalcogenides (i.e., V2 VI3 or Pn2 Ch3 (Pn = Pnictogen, Ch = Chalcogen) Compounds) from a Bonding Perspective. *Adv Mater* **31**, 1904316 (2019).

3. Yu Y, Cagnoni M, Cojocaru-Mirédin O, Wuttig M. Chalcogenide Thermoelectrics Empowered by an Unconventional Bonding Mechanism. *Adv Funct Mater* **30**, 1904862 (2020).

4. Schön C-F, *et al.* Classification of properties and their relation to chemical bonding: Essential steps toward the inverse design of functional materials. *Sci Adv* **8**, eade0828 (2022).

5. Zhu M, *et al.* Unique Bond Breaking in Crystalline Phase Change Materials and the Quest for Metavalent Bonding. *Adv Mater* **30**, 1706735 (2018).
