# Supplementary material for: Strong charge carrier scattering at grain boundaries of PbTe caused by the collapse of metavalent bonding
Source: Nat Commun. 2023 Feb 9;14:719. doi: 10.1038/s41467-023-36415-1 (PMC9911745; doi:10.1038/s41467-023-36415-1)
Supplement: Supplementary file 3 — Reporting Summary [file 41467_2023_36415_MOESM3_ESM.pdf]

naturereseach

Corresponding author(s): Yuan Yu; Oana Cojocaru-Mirédin; Matthias Wuttig

Lasing Reporting Summary

Nature Research wishes to improve the reproducibility of the work that we publish. This form is intended for publication with all accepted papers reporting claims of lasing and provides structure for consistency and transparency in reporting. Some list items might not apply to an individual manuscript, but all fields must be completed for clarity.

For further information on Nature Research policies, including our data availability policy, see Authors & Referees.

ü Experimental design

Please check: are the following details reported in the manuscript?

1. Threshold

Item	Yes	No	Comment
Plots of device output power versus pump power over a wide range of values indicating a clear threshold	☐	☒	we do not have such measurements

2. Linewidth narrowing

Item	Yes	No	Comment
Plots of spectral power density for the emission at pump powers below, around, and above the lasing threshold, indicating a clear linewidth narrowing at threshold	☐	☒	we do not have such measurements
Resolution of the spectrometer used to make spectral measurements	☐	☒	we do not have such measurements

3. Coherent emission

Item	Yes	No	Comment
Measurements of the coherence and/or polarization of the emission	☐	☒	we do not have such measurements

4. Beam spatial profile

Item	Yes	No	Comment
Image and/or measurement of the spatial shape and profile of the emission, showing a well-defined beam above threshold	☐	☒	we do not have such measurements

5. Operating conditions

Item	Yes	No	Comment
Description of the laser and pumping conditions *Continuous-wave, pulsed, temperature of operation*	☐	☒	we do not have such measurements
Threshold values provided as density values (e.g. W cm^{-2} or J cm^{-2}) taking into account the area of the device	☐	☒	we do not have such measurements

6. Alternative explanations

Item	Yes	No	Comment
Reasoning as to why alternative explanations have been ruled out as responsible for the emission characteristics *e.g. amplified spontaneous, directional scattering; modification of fluorescence spectrum by the cavity*	☐	☒	we do not have such measurements

7. Theoretical analysis

Item	Yes	No	Comment
Theoretical analysis that ensures that the experimental values measured are realistic and reasonable *e.g. laser threshold, linewidth, cavity gain-loss, efficiency*	☐	☒	we do not have such measurements

8. Statistics

Item	Yes	No	Comment
Number of devices fabricated and tested	☒	☐	The number of micro-devices fabricated and tested can be found in Fig. 2.
Statistical analysis of the device performance and lifetime (time to failure)	☐	☒	we do not have such measurements

March 2018
